# Characteristics of Human Endometrium-Derived Mesenchymal Stem Cells and Their Tropism to Endometriosis

**DOI:** 10.1155/2017/4794827

**Published:** 2017-07-06

**Authors:** Yan Cheng, Liru Li, Dejun Wang, Qiuyan Guo, Yanan He, Tian Liang, Liyuan Sun, Xiaojun Wang, Yulei Cheng, Guangmei Zhang

**Affiliations:** ^1^Department of Gynaecology, The First Affiliated Hospital of Harbin Medical University, Harbin, China; ^2^Precision Medical Center, The Third Affiliated Hospital of Harbin Medical University, Harbin, China; ^3^Department of Ultrasound of Obstetrics and Gynaecology, The First Affiliated Hospital of Harbin Medical University, Harbin, China; ^4^The State Key Laboratory of Veterinary Biotechnology, Harbin Veterinary Research Institute, Chinese Academy of Agricultural Sciences, Harbin, China; ^5^College of Science, Harbin Engineering University, Harbin, China

## Abstract

Human endometrial tissue has become an attractive source of mesenchymal stem cells (MSCs) for cell-based therapies because these MSCs can be easily harvested and have tumour tropism as well as reduced immunogenic and inflammatory properties. Our study aimed to obtain and characterise human endometrial mesenchymal stem cells (EMSCs) and assess their endometriosis tropism. EMSCs were successfully isolated from the endometrium of women undergoing laparoscopy for idiopathic infertility. The EMSCs presented a fibroblast-like morphology during culture. Flow cytometry analyses showed that the cells were positive for the specific stem cell markers CD73, CD90, CD105, CD166, and HLA-ABC (major histocompatibility complex class I (MHC I)) but negative for CD14, CD34, CD45, and HLA-DR (MHC II). Reverse transcription polymerase chain reaction results showed that the EMSCs expressed the stem cell marker OCT4. The EMSCs could differentiate into osteocytes, adipocytes, and chondrocytes under certain conditions. The EMSCs had a high tropism to endometriosis without tumourigenicity. This study enhances the possibility of using EMSCs as drug carriers in human cell-based therapies. Meanwhile, future research could also focus on developing targeted therapies for endometriosis.

## 1. Introduction

Mesenchymal stem cells (MSCs) have been explored as a promising vehicle candidate for cell-based targeted therapies [1–3]. MSCs are adult nonhaematopoietic stem cells that can be isolated from a variety of organs and tissues, such as the bone marrow, adipose tissue, umbilical cord, placenta, and amniotic fluid, among many other tissues [4–6]. MSCs exhibit such characteristics as high proliferation, self-renewal, multipotency, low immunogenicity, and nononcogenicity [6, 7]. MSCs have a special capacity for migrating to sites of inflammation, including tumours and ectopic endometrial lesions, and they have shown promise as a therapeutic vehicle for many types of diseases [8]. There have been studies on the antitumour effects of MSCs from the bone marrow and other sources [5, 8, 9], but few have focused on endometrial mesenchymal stem cells (EMSCs). Endometrial stem cells were first introduced by Prianishnikov in 1978 [10] and were separated from endometrial tissue by Chan et al. in 2004 [11]. EMSCs, which are now widely used, were identified by Gargett et al. in 2009 [12]. EMSCs have all the characteristics of MSCs. An increasing amount of evidence suggests that EMSCs can be used for regenerative medicine and that they can be used as an immune regulator to reduce inflammation [13–16]. However, their application in targeted therapies is seldom reported.

Endometriosis is a gynaecological disorder that affects 15–25% women [17] and is characterised by the extrauterine presence of activated endometrial tissue [18]. It is responsible for chronic pelvic pain, dyspareunia, dysmenorrhoea, and infertility in women of childbearing age [19, 20]. Although endometriosis is a benign disease, it bears many features similar to malignant disease, such as infiltration, migration, and recidivation [18, 21]. Traditional treatments include hormone therapy, invasive surgery, and a combination of both. Although all these treatments are more or less effective, the recurrence risk is still high [22, 23]. Therefore, there is a clear need for specific targeted therapies with more tolerable side effects as well as lower cost profiles and recurrence risk. Biological treatments are increasingly accepted by patients. Targeted therapies are treatments resulting from the blending of multidisciplinary technologies in medicine, and they represent an important research direction. MSC-based targeted drug delivery systems have a distinct advantage, that is, they have the unique characteristics of eosinophilic tumour sites, and compared with the platforms of other targeted drug delivery systems, MSCs can migrate to tumour sites [8] regardless of tumour size, location, or source. Due to the tumour tropism of MSCs, we aimed to study the endometriosis tropism of EMSCs, which may provide new strategies for the targeted treatment of endometriosis.

As such, we propose to investigate the characteristics of EMSCs and their tropism to endometriosis, thereby laying the groundwork for targeted endometriosis therapy.

## 2. Materials and Methods

### 2.1. Specimen Sources

Human endometrial tissues were obtained from eight women aged 28–35 (31.7 ± 2.9) years undergoing laparoscopy for idiopathic infertility in the First Affiliated Hospital of Harbin Medical University. Ectopic endometrium tissues were obtained from ten patients aged 21–42 (29.7 ± 6.5) years undergoing laparoscopy for endometriosis. Surgery was scheduled to occur during the late proliferative phase of the menstrual cycle, and endometrial tissue samples were collected at the time of surgery. The patients had not taken hormone treatments for 3 months prior to surgery, and they had been diagnosed with endometriosis or an endometrial disorder. This study was performed in accordance with China's national legislation and approved by the Ethics Committee of the First Affiliated Hospital of Harbin Medical University, and the methods were conducted in accordance with the approved guidelines. Informed written consent was obtained from all patients before their participation. All animal experiments were conducted using BALB/c nude mice aged approximately 6 weeks and weighing 18–20 g; these mice were obtained from Slack Company in Shanghai (numbers 2013001808618/2013001809469), and the animal experiments were conducted in strict accordance with the recommendations in the Guide for the Care and Use of Laboratory Animals of the Harbin Medical University Ethics Committee. The protocol was approved by the Committee on the Ethics of Animal Experiments of the Harbin Medical University. All efforts were made to minimise animal suffering. HESCs were purchased from the American Type Culture Collection. HSFs were independently preserved by the obstetrics and gynaecology laboratory in the First Affiliated Hospital of Harbin Medical University, and all cell protocols were approved by the Harbin Medical University Ethics Committee.

### 2.2. Isolation of EMSCs

Endometrium tissues were collected in ice-cold medium (DMEM/F-12 1 : 1) (Gibco, Grand Island, NY, USA) containing 10% foetal bovine serum (Gibco, Grand Island, NY, USA) and 1% antibiotic penicillin/streptomycin (Gibco, Grand Island, NY, USA). The samples were stored at 4°C and processed within 6 h. The endometrial tissues were minced and dissociated in 300 *μ*g/ml type III collagenase and 40 *μ*g/ml type I deoxyribonuclease for 60 min at 37°C [11].

Cell suspensions were filtered through a 70 *μ*M sieve (HEAD, Beijing, China) to remove glandular epithelial cells. The filtrates were then centrifuged at 800 rpm for 10 min at room temperature (RT). The isolated cells were cultured in the medium mention above at 37°C in 5% CO_2_. The medium was changed after 3 days to remove nonadherent cells [11].

### 2.3. Cell Culture and In Vitro Colony-Forming Assay

Freshly sorted cells were incubated at 37°C in 5% CO_2_, and the medium was changed every 3 days.

For the colony-forming assays, cells were seeded at very low seeding densities of 10–50 cells/cm^2^ onto fibronectin-coated (10 *μ*g/ml) 6-well plates and cultured in complete medium that was changed on day 7. The cells were expanded to allow colonies to form. Colonies were monitored by microscopy daily to ensure their derivation from single cells. Colonies of spindle-shaped EMSCs were selected and subcultured. Subsequently, the cells were washed with phosphate-buffered saline (PBS), and the medium was changed every 3 days until the cell population reached 90% confluence. Then, the cells were trypsinised (0.25% trypsin and 0.01% ethylenediaminetetraacetic acid) into a single-cell suspension. Cells were subcultured on new plates at a ratio of 1 : 2 and marked as passage 1 (P1) [12]. Cells from P3–P5 were used for the experiments.

### 2.4. Observation of EMSC Morphology

The morphology of EMSCs from different passages was observed by inverted light microscopy (Olympus, Japan).

### 2.5. RT-PCR

The expression of OCT4 in EMSCs was detected by RT-PCR. HESCs were used as the positive control, and HSFs were used as the negative control. Total RNA was isolated using TRIzol with the genomic DNA removed. One-step RT-PCR was performed using the following cycling conditions: 42°C for 1 h; 94°C for 15 min; 35 cycles of 94°C for 30 s, 57°C for 30 s, and 72°C for 30 s; and 72°C for 10 min. The PCR products were visualised on a 1% agarose gel with ethidium bromide [24]. The primer sequences are listed in Table 1.

### 2.6. Flow Cytometry

Cell surface markers specific for stem cells were assessed by flow cytometry, including CD73, CD90, CD105, CD166, CD14, CD34, CD45, HLA-ABC, and HLA-DR [12]. Cells from P3–P5 were collected and resuspended in PBS at a concentration of 2 × 10^4^ cells/20 *μ*l. Then, they were incubated with the following antibodies for 20 min at RT in the dark: mouse IgG1-PE, mouse IgG1-FITC, CD90-FITC, CD105-FITC, CD73-FITC, CD14-PE, CD34-FITC, CD45-FITC, HLA-ABC-FITC, and HLA-DR-PE (BD Biosciences, San Jose, USA). Then, the cells were washed with PBS and centrifuged at 800 rpm for 5 min. After the supernatant was discarded, the cells were resuspended with 300 *μ*l of PBS. The antibody-labelled cells were analysed with a BD FACSCalibur flow cytometer. The data were analysed using CellQuest Pro software, which was provided by the manufacturer.

### 2.7. Differentiation of EMSCs

The capacity of EMSCs to differentiate in vitro was evaluated. EMSCs were seeded at 1 × 10^5^/cm^2^ and cultured for 3 weeks in osteogenic differentiation medium (0.01 *μ*M 1*α*-25-dihydroxyvitamin-D3, 10 mM *β*-glycerophosphate, and 50 *μ*M ascorbate-2) (Sigma-Aldrich, St. Louis, MO, USA), adipocyte differentiation medium (500 *μ*M isobutyl-methylxanthine, 1 *μ*M dexamethasone, 10 *μ*M insulin, and 200 *μ*M indomethacin) (Sigma-Aldrich, St. Louis, MO, USA), or chondrogenic differentiation medium (6.25 g/ml insulin, 50 *μ*M ascorbate-2) (Sigma-Aldrich, St. Louis, MO, USA). Cells cultured in normal medium were used as the control. The medium was changed every 3 days. After 3 weeks, the cells in each group were stained with alizarin red, oil red O, and alcian blue (Sigma-Aldrich, St. Louis, MO, USA) to assess osteogenesis, adipogenesis, and chondrogenesis, respectively [25].

### 2.8. EMSC Migration towards Endometriosis In Vitro

The tropism of EMSCs to endometriosis in vitro was evaluated by examining the migration of EMSCs to ECECs isolated from the ectopic endometrial tissue of patients with ovarian endometriosis. EUECs were used as the control. The ECECs and EUECs were isolated by a method similar to that used for isolating EMSCs and were not filtered; P1 cells were used for experiments. EMSCs were seeded in Transwell chambers (8 *μ*m, 24 mm) (Corning, New York, USA) at a concentration of 5 × 10^4^ cells/well and cocultured with ECECs and EUECs seeded in a 6-well plate (Corning, New York, USA) at 37°C in 5% CO_2_ for 24 h. The numbers of cells that migrated through the polyester membrane were quantified by microscopy after being fixed in ice-cold 2% formaldehyde for 30 min and stained with 0.1% crystal violet for 10 min.

### 2.9. Tumourigenicity Analysis of EMSCs In Vivo

Mice were kept on a 12 h light/dark cycle and provided with sterile food and water; the mice were allowed to acclimate to specific pathogen-free (SPF) conditions prior to the experiments. The mice were randomly separated into 2 groups (*n* = 5 per group): the EMSC and ectopic endometrium tumourigenicity groups (positive control group). EMSC tumourigenicity models were established by injecting 0.2 ml of normal saline (NS) with 1 × 10^7^ cells/ml into the right subcutaneous scapular tissue and injecting the contralateral side with 0.2 ml of NS as the negative control. Ectopic endometrium tumourigenicity models were established by injecting NS with ectopic endometrium fragments 0.5 cm^3^ in size into the right subcutaneous scapular tissue and injecting the contralateral side with 0.2 ml of NS as the negative control [26]. Tumour formation was monitored daily in both groups. After 40 days, the mice were sacrificed by cervical dislocation. Both the left and right subcutaneous scapular tissues were collected. Macroscopic observation and H&E staining were used to assess tumour formation.

### 2.10. Tropism of EMSCs to Endometriosis In Vivo

Human ectopic endometrial tissues were obtained from patients with ovarian endometriosis as described above. Mice were maintained on a 12 h light/dark cycle and were provided with sterile food and water; the mice were allowed to acclimate to SPF conditions prior to the experiments. Twenty mice received a single subcutaneous injection of a 0.5 cm^3^ ectopic endometrium fragment in 0.2 ml of NS into their backs. Seven days later, when the endometriosis model was confirmed, the mice were randomly divided into two groups (*n* = 10 per group): the EMSC and control groups. The injections were performed with cells labelled by CM-Dil (Thermo Fisher Scientific, MA, USA). In the EMSC group, 5 × 10^6^ cells suspended in 0.3 ml of NS were administered intravenously into the tail vein. Mice in the control group were only injected with 0.3 ml of NS. The injections were performed weekly. The animals were sacrificed one week after the third injection; the ectopic endometriosis tissue sizes were measured; and the endometriotic lesions, livers, lungs, kidneys, and spleens of the two groups were collected for DAPI staining (Solarbio, Beijing, China) and red fluorescence analysis by confocal laser scanning microscopy [6]. For the immunohistochemistry experiment, the collected tissues were fixed in 10% paraformaldehyde for 24 h at RT and then dehydrated using a graded series of alcohol. The paraffin embedded tissue sections were mounted onto microscope slides and dewaxed and rehydrated before antigen retrieval in citric acid (pH 6.0) for 2 min with a pressure cooker. After cooling, the slides were washed with distilled water, incubated with the primary antibody (antivascular endothelial growth factor (VEGF) and anti-CD34) at RT for 1 h, and then rinsed in running water and PBST. The slides were then stained with secondary antibody-1 for 20 min and secondary antibody-2 for 30 min, washed in running tap water, stained with DAB, and then counterstained with haematoxylin. Finally, they were dehydrated and visualised using a microscope. The results were analysed with Image-Pro Plus (IPP) 6.0 software (Media Cybernetics, Maryland, USA).

### 2.11. Statistical Analysis

All statistical analyses were performed using SPSS 19.0 (SPSS Inc., Chicago, IL). Continuous variables are expressed as the mean ± standard deviation. Differences between groups were evaluated using the independent samples Student *t*-test. Differences were considered statistically significant at *P* < 0.05.

## 3. Results

### 3.1. Isolation, Culture, and Morphological Observation of EMSCs

**Figure 1:**
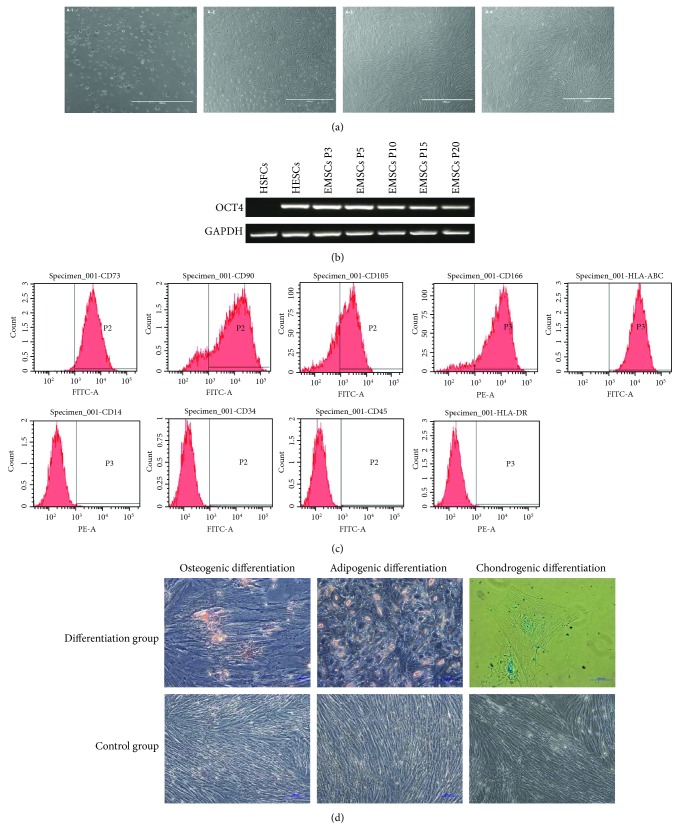
Isolation, cultivation, and identification of EMSCs. (a) (A-1) Nonadherent cells; (A-2) P1 cells; (A-3) P3 cells; (A-4) P5 cells. Scale bar = 1000 *μ*m. (b) Stem cell-specific marker OCT4 expression in P3, P5, P10, P15, and P20 EMSCs. OCT4 was positively expressed in EMSCs, and the OCT4 expression shown a gradually decreasing trend with increasing passage number. (c) Immunophenotypes of EMSCs. The flow cytometry analysis results showed that the EMSCs positively expressed CD73, CD90, CD105, and CD166 but negatively expressed CD14, CD34, and CD45. The results also showed that the EMSCs expressed HLA-ABC (MHC I), but not HLA-DR (MHC II). (d) EMSC differentiation in vitro. The staining results of EMSCs after being cultured in different induction media.

EMSCs were successfully isolated through the centrifugal adherence method from endometrium tissues. The first clones of adherent cells appeared 5–7 days after the initial plating. Primary EMSCs presented a short polygonal or fusiform morphology, which gradually changed into a fibroblast-like spindle shape with increasing passages. As shown in Figure 1(a), with rapid cell proliferation, cells from passages 3–5 (P3–P5) exhibited a relatively uniform, long spindle shape and a swirling arrangement, similar to the features of bone marrow MSCs.

### 3.2. Stem Cell-Specific Marker OCT4 Expression in EMSCs

Human embryonic stem cells (HESCs) were used as the positive control, and human skin fibroblasts (HSFs) were used as the negative control. The reverse transcription polymerase chain reaction (RT-PCR) analysis showed positive OCT4 expression in EMSCs and that the OCT4 expression shown a gradually decreasing trend with increasing passages (Figure 1(b)).

### 3.3. Immunophenotypes of EMSCs

According to the flow cytometry analysis, we discovered that the EMSCs were positive for CD73, CD90, CD105, and CD166 but negative for CD14, CD34, and CD45, indicating their MSC phenotype. Our results also showed that the EMSCs weakly expressed HLA-ABC (major histocompatibility complex class I (MHC I)) and expressed no HLA-DR (MHC II), indicating the low immunogenicity of the EMSCs (Figure 1(c)).

### 3.4. EMSC Differentiation In Vitro

The negative control cells were not stained by alizarin red after being cultured in complete medium for three weeks. The appearance of EMSCs obviously changed after being incubated in osteogenic induction medium for 21 days, and the cells were capable of osteogenic differentiation, as indicated by the observation of calcium nodules after alizarin red staining (Figure 1(d)).

After being cultured in complete medium for three weeks, the negative control cells were not stained by oil red O. As indicated by lipid droplets visualised by oil red O staining, the EMSCs could differentiate into adipocytes in vitro after 21 days of induction (Figure 1(d)).

The negative control cells were not stained by alcian blue after being cultured in complete medium for three weeks. After 21 days of culture in chondrogenic differentiation medium, the EMSCs showed positive alcian blue staining (Figure 1(d)), which indicated their capacity to differentiate into chondrocytes under specific conditions.

### 3.5. EMSCs Migrated towards Endometriosis In Vitro

**Figure 2:**
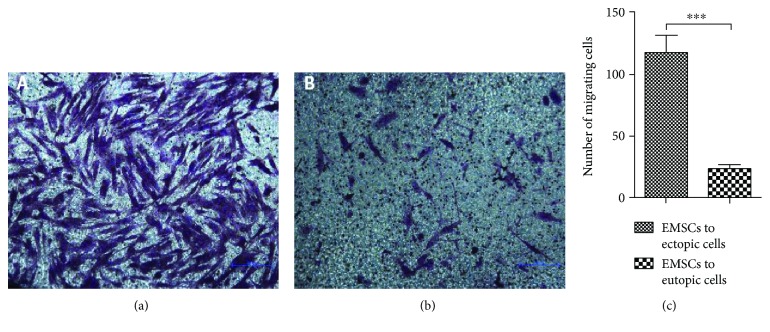
EMSC migration towards endometriosis in vitro. (a) EMSCs migrated towards ectopic endometrial cells in vitro. The cells were counted by microscopy. Scale bar = 200 *μ*m. (b) EMSCs migrated towards EUECs in vitro. The cells were counted by microscopy. Scale bar = 200 *μ*m. (c) Diagram of EMSC tropism to endometriosis in vitro. The EMSCs revealed a significantly higher tropism to the ectopic endometrial cells of patients with endometriosis than to the control eutopic cells. The error bars, standard deviation, and *P* values were determined using the two independent samples *t*-test; ^∗∗∗^*P* < 0.001.

A Transwell culture system was used to evaluate the tropism of EMSCs towards endometriotic cells in vitro. As shown in Figure 2, EMSCs (117.40 ± 12.42) revealed a significantly higher tropism to the ectopic endometrial cells (ECECs) of patients with endometriosis compared to eutopic endometrial cells (EUECs) (23.20 ± 3.19, *P* < 0.0001).

### 3.6. EMSCs and Ectopic Endometrium Tumourigenicity Analysis

**Figure 3:**
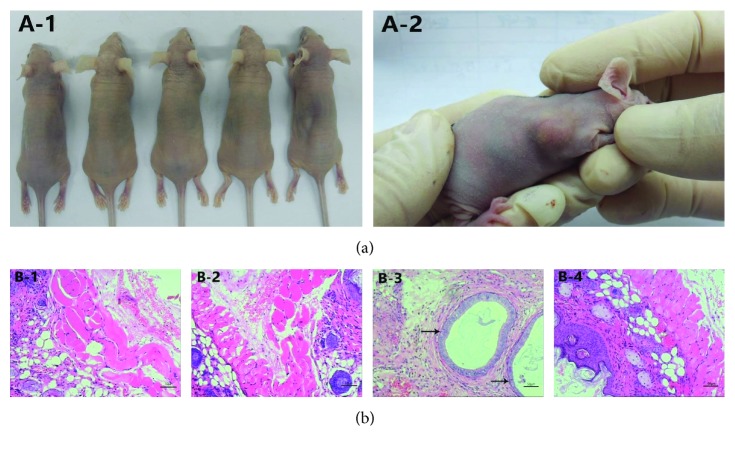
EMSCs and ectopic endometrium tumourigenicity. (a) Macroscopic observation. (A-1) Absence of tumour formation from inoculated EMSCs. (A-2) Lesion formation from ectopic endometrium inoculation. (b) H&E staining. (B-1) EMSC injection site after H&E staining in the EMSC group. (B-2) NS injection site after H&E staining in the EMSC group. (B-3) Ectopic endometrium injection site after H&E staining. Arrow indicates endometrial glands. (B-4) NS injection site after H&E staining in the ectopic endometrium group.

To investigate whether EMSCs could form tumours or cause significant side effects in vivo, 6-week-old nude mice were subcutaneously inoculated with 1 × 10^7^ EMSCs or an ectopic endometrium fragment (*n* = 5 per group). Three days after the EMSCs were injected, the right side of the nude mice showed soft skin rashes at the injection site, while no skin rashes were observed on the left site. Two days later, the skin rashes disappeared. Neither distinct weight loss nor any symptoms of poor health were observed during the experiment. The mice were maintained for 40 days before being sacrificed, and no visible solid tumours were found around the injection sites by macroscopic observation in the EMSC group (Figure 3(a), A-1). Subcutaneous tissues were collected from both the scapular EMSC and saline injection sites. The pathological diagnosis made via haematoxylin and eosin (H&E) staining confirmed the absence of tumour generation (Figure 3(b), B-1). No tumours formed on the left side (Figure 3(b), B-2). These results suggested that the EMSCs had no tumourigenicity and could be safely used in vivo.

**Figure 4:**
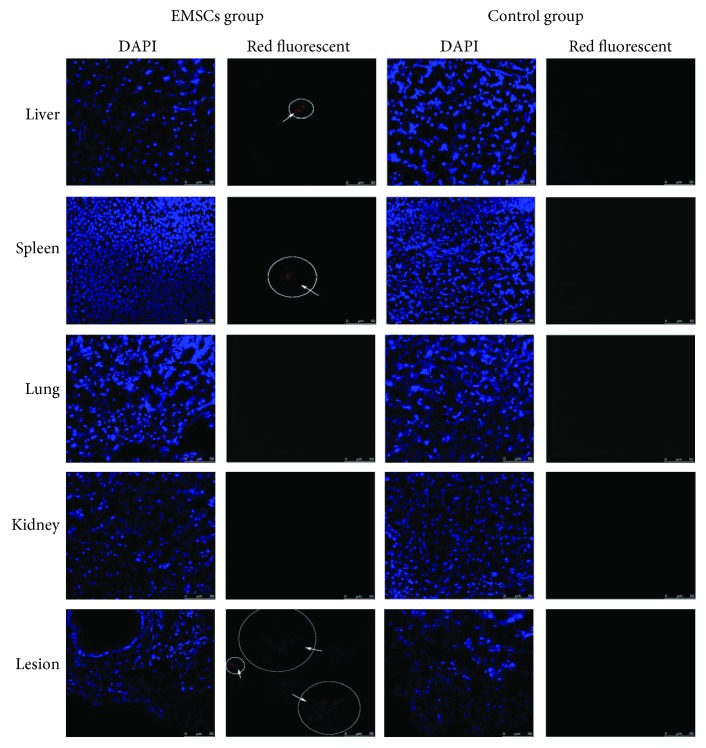
Tropism of EMSCs to endometriosis in vivo. Confocal laser scanning microscopy was used to detect red fluorescence in liver, spleen, lung, kidney, and lesion tissue samples from both groups. The amplified view of EMSCs migrated to liver, spleen, and endometriotic lesion were shown as circles with arrows.

However, two days after ectopic endometrium fragment injection, the right side of the 5 nude mice showed soft skin rashes at the injection site, while no skin rashes appeared on the left side. Two days later, the skin rashes on the right side of the nude mice were still present, and no tumours had formed at the left scapular injection site. Five days after the injection, slightly soft round masses were visible on the right side of the 5 nude mice, and these lesions increased in size over time. At 40 days after the injection, the mice were sacrificed, and subcutaneous tissues were collected from both the scapular ectopic endometrium fragment and saline injection sites. By macroscopic observation (Figure 3(a), A-2) and H&E staining (Figure 3(b), B-3), we found that endometrial-like tissues had been generated on the right side of the 5 nude mice, while no tumours formed on the left side (Figure 3(b), B-4). These results indicated that the ectopic endometrium fragment had tumourigenicity.

### 3.7. Tropism of EMSCs to Endometriosis In Vivo

1,1′-Dioctadecyl-3,3,3′,3′-tetramethylindocarbocyanine perchlorate-Dil- (CM-Dil-) labelled cells were detected by their red florescence. EMSCs were observed to be mainly gathered in endometriotic lesions (Figure 4), and a small number of EMSCs were observed in the livers and spleens. These results suggest that the EMSCs had a tropism to endometriotic lesions rather than normal tissues, indicating their potential for targeted endometriosis therapy.

### 3.8. The Effect of EMSCs on Endometriotic Lesions in the Mouse Model

To investigate the effect of EMSCs on endometriotic lesions in our mouse model, the ectopic endometriosis tissue sizes of each group were measured after the lesions were collected. The score of each lesion was calculated according to the sum of the length and width. No significant differences were observed between the EMSC group (16.60 ± 4.72 mm) and the control group (16.90 ± 3.47 mm, *P* = 0.912; Figure S1 available online at https://doi.org/10.1155/2017/4794827).

Angiogenesis in the endometriotic lesions of each group was evaluated by the expression of the angiogenesis-associated protein VEGF. VEGF expression was analysed by quantifying the density of the positively stained cells with IPP 6.0. No significant differences were observed between the EMSC group (0.0262 ± 0.0031) and the control group (0.0276 ± 0.0026, *P* = 0.321; Figure S2). In addition, the microvascular density (MVD) was assessed using the CD34 antibody. The number of CD34-positive cells was counted at low magnification. No significant differences were observed between the EMSC group (27 ± 5) and the control group (28 ± 7, *P* = 0.831; Figure S2).

## 4. Discussion

Endometriosis is a benign, chronic gynaecological disease that shares several characteristics with invasive cancer types, and its global incidence is rising [17, 18]. Although medical and surgical therapies have been applied for the treatment of endometriosis, there is still no ideal therapeutic method for the disease. Hormonal treatments have many side effects and can only temporarily inhibit the growth of endometriotic lesions versus eliminating them. Traditional surgical approaches can excise ectopic lesions, but the disease recurs in many patients [22, 23]. The deficiencies of the currently available treatments are due to the lack of therapeutic strategies against the pathogenesis of the condition. Therefore, exploring new treatments for endometriosis is an urgent matter. MSCs are currently one of the most promising candidates for cell-based targeted therapies due to their low immunogenicity, nontumourigenicity, and tumour tropism [1, 8, 27]. The utilisation of EMSCs has been studied in regenerative medicine [13, 28]. However, to the best of our knowledge, their application as a gene therapy vector has yet to be reported.

We isolated EMSCs from women without endometrium-associated diseases, such as endometriosis, adenomyosis, and endometrial hyperplasia. In our study, the cells we separated and cultured exhibited a long spindle shape and a swirling arrangement, similar to the features of other MSC types, as described elsewhere [6, 29]. The observed OCT4 expression and the gradual decrease in OCT4 expression with increasing passages indicated that the EMSCs we cultured had stem cell characteristics [24]. The cells also expressed high levels of CD73, CD90, CD105, and CD166, which are the main stem cell markers, but they lacked CD14, CD34, and CD45 expression; these results were in agreement with those of previous studies [6, 12, 30]. However, as there are no specific markers to confirm the identification of MSCs, further tests are needed to be performed. The cells were capable of differentiating into adipocytes, osteocytes, and chondrocytes in vitro, which indicated their multipotency [12, 31]. According to the definition provided by the Committee of the International Society for Cellular Therapy, MSCs are multipotent cells that are adhere to plastic; that express CD73, CD90, and CD105 instead of CD14, CD34, CD45, and HLA-DR; and that must be able to differentiate into osteoblasts, adipocytes, and chondroblasts in vitro [32]. Accordingly, the above results indicate that the cells we isolated and cultured were EMSCs. Moreover, EMSCs exhibit a high proliferative capacity, multidirectional differentiation, and the potential for expansion. These characteristics indicate that EMSCs may be potentially be used for tissue reconstruction. In addition, as the EMSCs expressed the MHC I molecule HLA-ABC but did not express the MHC II molecule HLA-DR, the cells showed low immunogenicity. Thus, EMSCs can be used for in vivo experimentation without the occurrence of immune rejection.

After the mice were injected with EMSCs, visual inspection and H&E staining revealed no tumour formation, suggesting that EMSCs could be safely used in vivo without causing tumour formation. However, whether EMSCs may cause tumours after being injected into the blood vessels or other areas requires further study.

Much of the available literature demonstrates that MSCs have a tropism to tumour tissues and sites of inflammation [8, 33]. Thus, as MSCs, EMSCs are capable of tumour and inflammatory tropism and of participating in endometriosis development; thus, they could be used in cell-based targeted therapies. In our study, when endometriotic cells were cocultured with EMSCs, these stem cells revealed a significant tropism to endometriotic cells. Similarly, in vivo experiments were performed to determine whether EMSCs had endometriosis tropism by injecting red fluorescence-labelled EMSCs into the tail vein of mice in an endometriosis model. Then, red fluorescence was detected by confocal laser scanning microscopy. The EMSCs were found mainly to have homed to the endometriotic lesions, and a small number of cells were observed in the livers and spleens. This phenomenon may occur due to the tropism of MSCs to sites of tissue damage and the inflammation or tumour microenvironment, which has been shown by many studies. Endometriotic lesions may produce such substances as proinflammatory molecules to attract EMSCs.

Given that there was no significant difference between the endometriotic lesion sizes of each group, we demonstrated that EMSCs do not affect the growth of endometriotic tissues. According to “Sampson's Theory,” which states that attachment-aggression-angiogenesis (“3A”) is the “trilogy” of the development of endometriosis [34], angiogenesis has become a universally accepted pathogenesis mechanism for endometriosis [35]. VEGF is a key mediator of angiogenesis. In previous studies, the increased expression of VEGF facilitates the survival of ectopic tissue in the peritoneal cavity and the development of endometriotic lesions [36]. Therefore, we detected the effect of EMSCs on angiogenesis in our xenograft model by examining the expression of VEGF in the lesion but found no significant reduction compared with the control group. In addition, we also evaluated the effect of EMSCs on angiogenesis in our xenograft model by examining MVD in the lesion and found no significant reduction in blood vessels compared with the control group. These results indicate that EMSCs do not affect angiogenesis in endometriotic lesions. However, other potential effects of EMSCs on endometriosis or whether EMSCs can suppress endometriosis after being transformed by transgenic manipulation requires further research.

## 5. Conclusion

In conclusion, with their low immunogenicity, nontumourigenicity, and endometriosis tropism, EMSCs have potential as a drug delivery system for targeted endometriosis therapies.

## Supplementary Material

Figure S1. The effect of EMSCs on the sizes of the endometriotic lesions. The ectopic endometriosis tissue sizes of each group were measured after the lesions were collected. The score of each lesion was calculated according to the sum of the length and width. Figure S2. The effect of EMSCs on angiogenesis in the endometriotic lesions. The expression of VEGF and CD34 were examined using immunohistochemistry.

## Figures and Tables

**Table 1:** Primer sequences used in the study.

Genes	Primer sequences	Products (bp)
OCT4	F: 5′-CGTGAAGCTGGAGAAGGAGAAGCTG-3′	247
	R: 5′-CAAGGGCCGCAGCTTACACATGTTC-3′	
GAPDH	F: 5′-GCTTGTCATCAATGGAAATCCC-3′	360
	R: 5′-TCCACACCCATGACGAACATG-3′	
