# Supplementary figures and images for: Characteristics of Human Endometrium-Derived Mesenchymal Stem Cells and Their Tropism to Endometriosis
Source: Stem Cells Int. 2017 Jul 6;2017:4794827. doi: 10.1155/2017/4794827 (PMC5518492; doi:10.1155/2017/4794827)

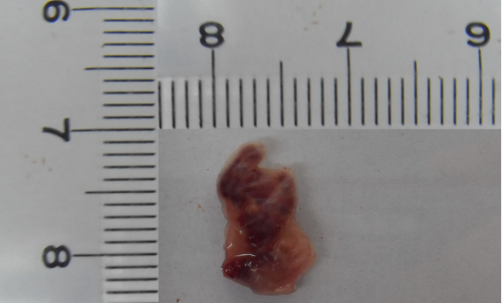

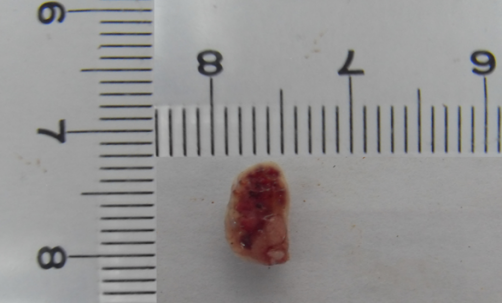

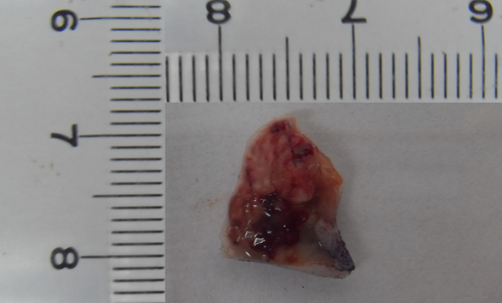

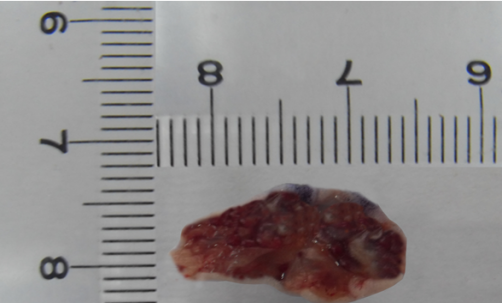

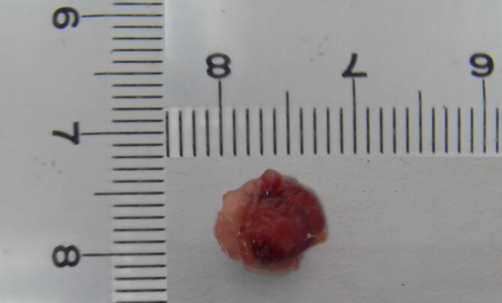

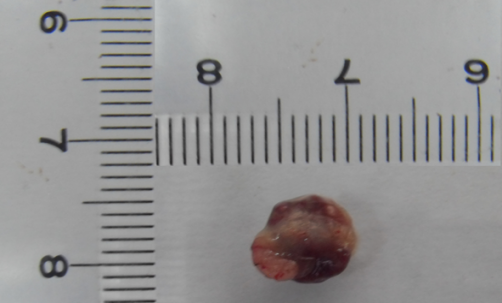

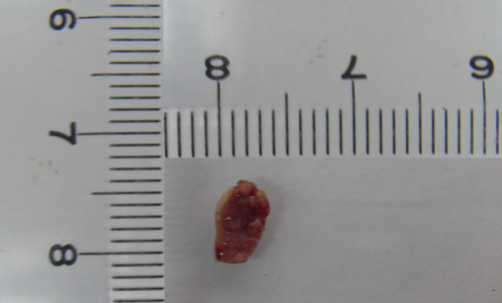

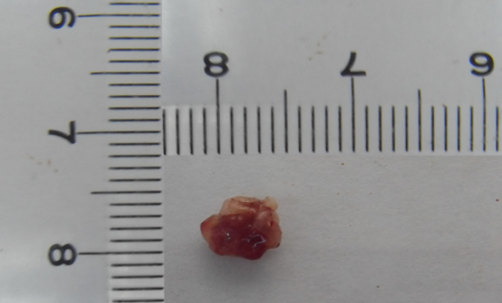

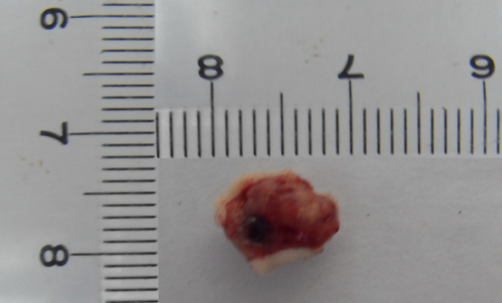

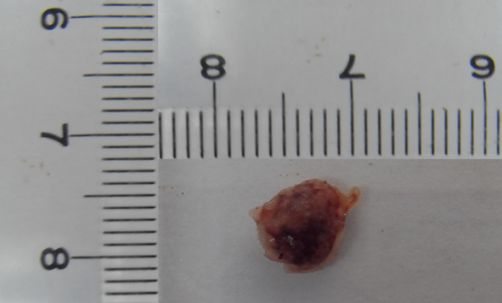


**EMSCs**

**Control**

Supplement: Supplementary file 1 — Figure S1. The effect of EMSCs on the sizes of the endometriotic lesions. The ectopic endometriosis tissue sizes of each group were measured after the lesions were collected. The score of each lesion was calculated according to the sum of the length and width. Figure S2. The effect of EMSCs on angiogenesis in the endometriotic lesions. The expression of VEGF and CD34 were examined using immunohistochemistry. [file 4794827.f1.docx]

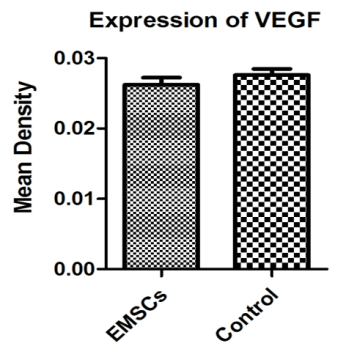

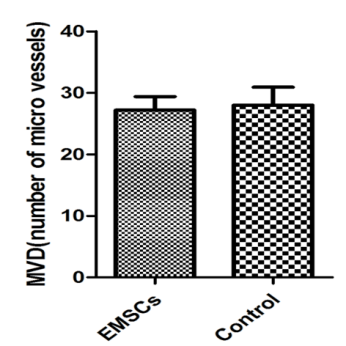

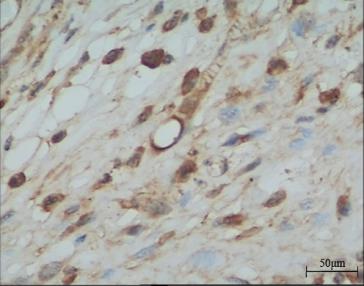

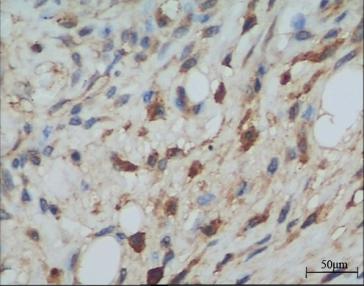

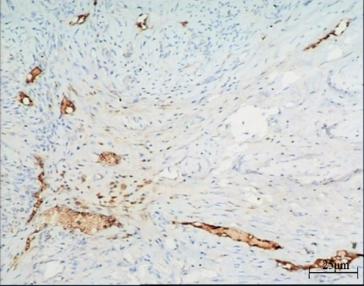

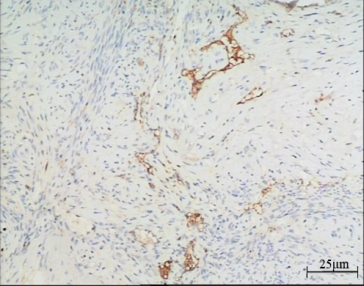


**Control**

**EMSCs**

**VEGF**

**MVD**

Supplement: Supplementary file 2 [file 4794827.f2.docx]
